# A robust lightweight deep learning network with deformable convolutions for efficient segmentation of optical coherence tomography images

**DOI:** 10.3389/fdgth.2026.1892323

**Published:** 2026-10-09

**Authors:** Mehmood Nawaz, Syed Muhammad Tariq Abbasi, Sheheryar Khan, Muhammad Ameer Hamza, Khadija Bibi, Syeda Aimen Abbasi, Muhammad Daud, Muhammad Asim, Wu Yuan

**Affiliations:** 1 Department of Data Science and Digital Innovation, The Hong Kong Shue Yan University, Hong Kong SAR, China; 2 Department of Biomedical Engineering, The Chinese University of Hong Kong, Hong Kong SAR, China; 3 Division of Science, Engineering, and Health Studies (SEHS), School of Professional Education and Executive Development, The Hong Kong Polytechnic University, Hong Kong SAR, China; 4 Department of Science, English Schools Foundation College, Sha Tin, Hong Kong SAR, China; 5 Department of Agriculture, Bahauddin Zakariya University, Multan, Pakistan

**Keywords:** deformable convolutional networks, lightweight architecture, optical coherence tomography, retinal and endoscopic imaging, semantic segmentation

## Abstract

**Introduction:**

Manual segmentation and evaluation of optical coherence tomography (OCT) images are difficult and rely on specialist training, which limits the output in a busy clinic. While automatic segmentation can ease this burden, the deep models that currently lead the field tend to be too heavy for routine deployment on standard clinical hardware. A further obstacle is the unusual geometry of OCT data: instead of compact objects on a background, the structures of interest form long, undulating tissue strata that extend horizontally across the whole frame, and this layout is poorly matched to convolutional kernels designed for ordinary photographs.

**Methods:**

We address both issues with LiDeOCTNet, a compact segmentation backbone tailored to the layered nature of OCT. Within a reduced UNet, we introduce learnable sampling offsets so that each kernel can reshape itself locally and trace the curved, irregular interfaces between tissues. To assess the design, we evaluate the network on three OCT collections covering retinal as well as endoscopic acquisitions.

**Results:**

Across all three, LiDeOCTNet surpasses a parameter-matched UNet baseline and approaches the accuracy of much heavier benchmarks such as TransUNet, despite using about 750× fewer parameters and running roughly 7.4× faster at inference.

**Discussion:**

Together these properties point to LiDeOCTNet as a practical option for real-time OCT analysis pipelines, including embedded and resource-limited devices.

## 1 Introduction

Optical coherence tomography (OCT) captures deep, micrometer-scale views of tissue architecture without the need for injections or direct contact. In the ophthalmic field, it is the standard method for evaluating the layered anatomy of the eye; in this context, the reliability of tissue segmentation is a cornerstone of diagnostic and therapeutic success (1, 2). More recently, however, OCT has moved into endoscopic applications, utilizing catheters to probe the internal linings of the airway and gut (3–6). Manually detecting the special tissues in OCT images are difficult, such as the muscularis of the bronchial wall or the mucosal layers of the colon (5, 6). Yet, relying on human experts to mark these boundaries is inefficient, as the process is labor-intensive and prone to subjective disagreement (7, 8). Modern OCT scanners work very fast, which makes the problem even worse. This shows we really need an automatic system that can keep up with all the data in busy clinics.

The task which assigns a class label to every pixel of an image is usually called dense or semantic prediction. It is now a foundational computer-vision operation (6, 9–11). Convolutional neural networks (CNNs) (12) have been the principal driver of recent progress, and dense prediction now powers a wide spectrum of medical and robotic systems, from intra-operative tool tracking (13) to autonomous navigation in clinical settings (14–16) and the automated reading of diagnostic scans (17, 18). CNNs have likewise become the default choice for OCT segmentation (19–21). The most accurate of these systems, however, typically rest on deep stacks of dilated convolutions in order to enlarge the effective receptive field (22), and this comes at the price of high memory consumption and a heavy computational budget that is at odds with real-time use, especially when the OCT system itself is operating at a high frame rate. Encouragingly, recent reports indicate that an architecture deliberately tailored to a specific task can deliver layer-level metrics and live structural views during acquisition without sacrificing accuracy (6).

One feature that sets OCT apart from natural imagery is its cross-sectional, depth-resolved acquisition mode (1, 23). The structures of clinical interest stretch across the entire lateral extent of each scan and are separated by interfaces that fluctuate in shape and thickness. This stands in sharp contrast to typical computer-vision imagery, in which the salient objects sit in compact, well-bounded regions of the frame (24–27). The mismatch produces a category of segmentation difficulty that off-the-shelf pipelines were never designed to handle.

Regular CNNs only look at small patches of an image at a time. For wider view, these models use a lot of layers, which makes the whole process more complicated (28, 29). While spatial context can be expanded via pooling or strided operations, these methods involve an inherent trade-off with image resolution. Even with subsequent upsampling, the loss of fine-scale architectural detail is often irreversible. This limitation is particularly damaging when processing OCT scans. In these OCT images, you cannot detect a single dot just by looking at that one spot. To solve this issue, we have to see how all the layers of tissue fit together. Most regular AI tools miss this because they only look at small areas at a time instead of the big picture (8, 30, 31).

Currently, the AI tools have capability to see the whole OCT image without losing any detail. The downside is that this makes the software much larger and really slows things down (29). The non-local module proposed by Wang (32) attempts to model such long-range dependencies explicitly, yet its rigid form and computational footprint make it impractical for compact, real-time deployments. Dilated convolutions (33) broaden the spatial coverage of a fixed-shape kernel without adding parameters, but because the kernel geometry itself remains static, they are better matched to coarser tasks like image-level classification than to the fine boundary tracing required here. Vision Transformers (ViTs) (34) approach the problem from a different angle, using self-attention to capture extended dependencies, but their substantial data and compute demands sit awkwardly with the relatively small annotated corpora that characterise OCT research (35–40).

A handful of very recent papers have started to tackle the lightweight requirement head-on. He et al. (41) put forward LightReSeg, a transformer-augmented retinal layer segmentation network that combines multi-scale feature extraction with an asymmetric attention module to keep parameter counts low while preserving accuracy. In a similar spirit, Qi et al. (42) proposed LKMU-Lite, a U-shaped network for retinal fluid segmentation that builds in a decoupled large-kernel attention block, and Gao et al. (43) reported FRNet V2, a full-resolution CNN for OCTA vessel segmentation that achieves competitive accuracy with more than 90% reduction in parameters. Collectively, these results show that compactness and accuracy do not have to be traded against each other for OCT data. Most of these designs, however, remain focused on retinal images and rely on attention or transformer components, leaving the question of shape-adaptive convolutions in OCT largely unexplored.

The architectural problem we set out to solve, then, is to find a network that is intrinsically aligned with the wide, irregular tissue bands found in OCT, while remaining small enough for low-latency operation. The dominant trend in earlier OCT segmentation work (44, 45) has instead been to push accuracy upward by enlarging the model, with relatively little concern for runtime efficiency. As a result, a compact, OCT-specific design that delivers fast quantitative measurements directly from raw scan data has so far been missing. The present paper aims to fill this gap with LiDeOCTNet, a lightweight segmentation network that pairs a slimmed-down UNet backbone with deformable convolution kernels whose sampling positions adapt to the surrounding tissue geometry. The complete architecture is illustrated in Figure 1.

**Figure 1:**
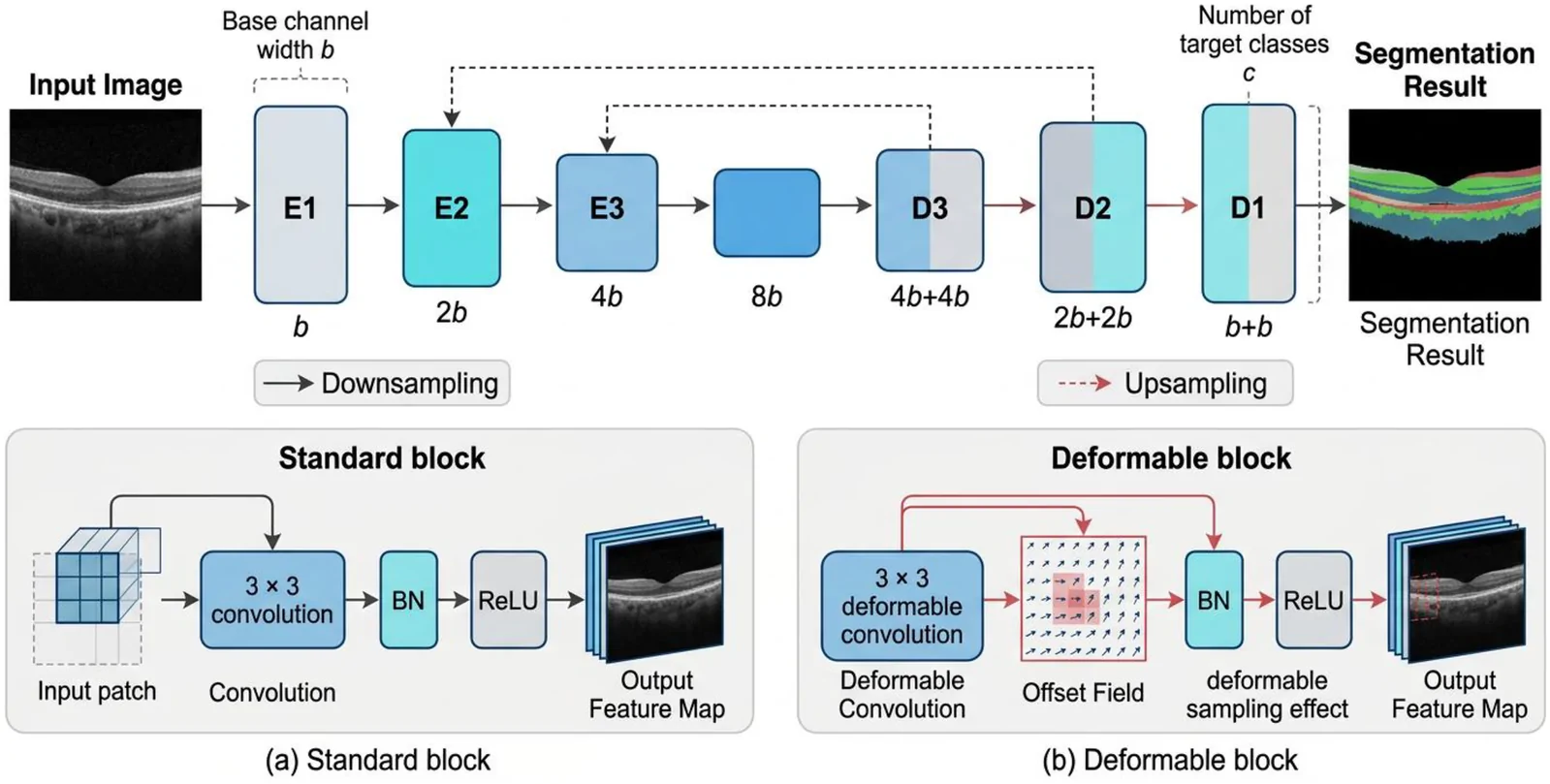
Overview of LiDeOCTNet. The network is built on a three-level UNet backbone with mirrored downsampling and upsampling branches. Two kinds of building block populate the network: **(a)** the standard block, where a 3 × 3 convolution is followed by batch normalisation (BN) and a ReLU activation, and **(b)** the deformable block, in which the 3 × 3 kernel is swapped for a deformable variant while BN and ReLU are retained. The hyperparameter b controls the base channel width and is the main lever for scaling the model; values of b from 4 up to 64 were used in our experiments. The variable c denotes the number of foreground tissue classes the network must predict.

Deformable convolutions (46) have been used previously by DUNet (47), but in a very different setting: that work targeted retinal vessel segmentation in fundus photography, where the structures of interest are fine, twisted tubular networks rather than the wide horizontally banded tissues seen in OCT. For our OCT segmentation problem, LiDeOCTNet uses fewer deformable blocks and a noticeably smaller backbone than DUNet yet still attains comparable accuracy. The experimental results in later sections demonstrate that LiDeOCTNet outperforms a UNet of equal size, and additionally that it remains competitive with three substantially heavier reference architectures: TransUNet (35), which couples a transformer encoder to a UNet-style decoder; DefEdNet (48), which integrates deformable kernels throughout the encoder; and BlazeNeo (49), a lightweight design built on the HarDNet backbone. TransUNet exceeds 100 million parameters and is therefore impractical for real-time scenarios. DefEdNet places deformable kernels at every encoder level along with a feature-fusion module, increasing complexity for only a modest accuracy gain on OCT data. BlazeNeo derives from the Harmonic DenseNet (HarDNet) family (50) with a reduced number of features scales but inherits the dense layer-to-layer connectivity of DenseNet (51), which still prevents it from reaching the compactness needed for high-throughput OCT processing.

The principal contributions of this study can be summarised as follows.

- We introduce LiDeOCTNet, a parameter-efficient architecture aimed at delineating tissue layers with irregular boundaries while keeping computational overhead low. The design is validated on both retinal and endoscopic OCT collections and compared against a standard UNet as well as several competitive recent baselines.
- We map out the functional parameter range of LiDeOCTNet, identifying the smallest model size at which segmentation behaviour remains stable.
- We perform a systematic ablation of where in the encoder–decoder hierarchy the deformable block should live, and quantify how its depth affects boundary accuracy.
- We propose a dataset-level complexity index that captures the two principal difficulties in OCT segmentation, namely boundary tortuosity and inter-class pixel imbalance, and we use it to motivate a tailored loss formulation.

The remainder of the paper proceeds as follows. Section 2 introduces LiDeOCTNet together with its building blocks, the datasets used, the preprocessing pipeline, the evaluation criteria, and the training protocol. Section 3 presents the experimental study, covering ablations and comparisons with state-of-the-art baselines. Section 4 provides a focused discussion of deformable block placement, network capacity, dataset complexity, and loss-function design, and Section 5 summarises the conclusions and points to future directions.

## 2 Method and materials

### 2.1 Lightweight deformable UNet model

LiDeOCTNet brings together two well-established ingredients: the encoder–decoder design pioneered by UNet (52) and the spatially adaptive sampling proposed in deformable convolutional networks (46). Its task is to delineate tissue layers whose boundaries are irregular in shape. The architecture is laid out in Figure 1. Following the canonical U-shape, an encoding branch progressively reduces the spatial resolution of the feature representations, while a mirrored decoding branch progressively restores it. At each resolution stage, the elementary block is a convolution followed by batch normalisation (53) and a ReLU non-linearity (54). Selected variants of LiDeOCTNet substitute one or more of these standard blocks for deformable counterparts, either inside the encoder, inside the decoder, or in both branches. A deformable block preserves the BN and ReLU stages but replaces the fixed-shape convolution with a deformable kernel of the same spatial footprint.

Within the encoder, downsampling is realised by setting the stride of the first convolution in each successive block to two. On the decoder side, bilinear interpolation upsamples the feature maps back to their original resolution. Skip connections concatenate encoder and decoder feature maps that share the same spatial scale. Compared with the four-level UNet that is common in the literature, LiDeOCTNet uses three resolution levels; the additional flexibility introduced by the deformable kernels compensates for the missing stage. Overall network capacity is governed by a single hyperparameter, the base channel count b (see Figure 1).

The input to the network is a single-channel grey-scale OCT image I, and the output is a pixel-level label map. Each location is assigned either to one of the c tissue layer classes or to the background class (label 0), so the full label set is C = {0, 1, …, c}. As training progresses, the deformable offset fields gradually adapt to the geometric shape and spatial extent of each tissue class, fusing low-level edge cues with higher-level structural context and so enabling tighter boundary delineation.

### 2.2 Deformable convolution kernel

Tracking the irregular contours of tissue interfaces is among the more demanding aspects of OCT segmentation. LiDeOCTNet overcomes these hurdles by leveraging deformable convolution layers (46) integrated into specific processing blocks (Figure 1b). Conventional kernels typically extract features from a uniform, static grid; however, the deformable approach introduces learnable offsets that displace these sampling points from their nominal arrangement. This lack of constraint allows the kernel's receptive field to “morph' and align itself with the actual anatomical contours of the tissue. Since this sampling pattern is conditioned on the kernel's spatial center, its configuration adapts as it moves through various tissue types. For an input feature centred at position *x*(*p*), the corresponding output *y*(*p*) is given by Equation (1):

$$y\left(p\right)\;=\;\overset{K}{\underset{k=1}{\sum }}\;w_{k}\;\cdot \;x\left(\;p\;+\;p_{k}\;+\;\Delta p_{k}\right)\;$$ (1)

in which *p*ₖ denotes the nominal relative position of the *k*-th node, *w*ₖ is its weight, *K* is the total number of sampling nodes, and Δ*p*ₖ is the learned offset that displaces node *k* from its nominal location (46). The mechanism allows the network to dynamically retune its sampling pattern to the diverse shapes of OCT tissue classes. A useful side benefit emerges when the kernel covers several tissue layers at once: the consistent anatomical stacking that characterises every OCT acquisition can then be exploited to determine which layer the central pixel belongs to.

Because deformable kernels share the structural form of standard kernels and only contribute additional offset parameters, they slot into any existing CNN architecture without disturbing the rest of the design. Within LiDeOCTNet, deformable kernels are deployed at carefully selected positions inside the lightweight UNet backbone, and the experiments below identify which placements yield the largest performance benefit.

### 2.3 Network implementations

To identify the minimal number of deformable blocks required for dependable OCT segmentation, we constructed five variants of LiDeOCTNet (Figure 2). Across this family, deformable blocks appear in either the encoding branch, the decoding branch, or both branches simultaneously. When deformable blocks populate both branches, the resulting variants are labelled LiDeOCTNet-ED1, ED2, and ED3, with the index marking whether the blocks sit at the deepest, intermediate, or shallowest resolution stage of the U-shaped hierarchy. This collection of configurations enables a controlled study of whether the most spatially compressed levels of the network are the most fruitful sites for deformable kernels in OCT.

**Figure 2:**
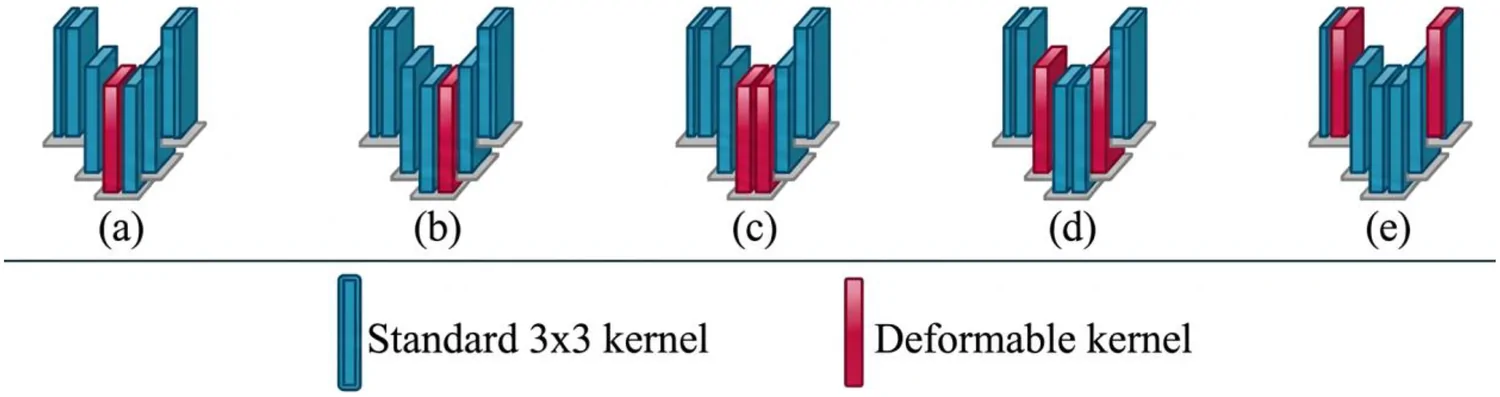
The five LiDeOCTNet variants examined in this work: **(a)** LiDeOCTNet-E, with one deformable block at the deepest encoder stage; **(b)** LiDeOCTNet-D, with one deformable block at the deepest decoder stage; **(c)** LiDeOCTNet-ED1, with deformable blocks at both deepest encoder and decoder stages; **(d)** LiDeOCTNet-ED2, where the deformable blocks sit at the intermediate level; and **(e)** LiDeOCTNet-ED3, where they are placed at the outermost (highest-resolution) level. The numerical suffixes 1, 2 and 3 thus index increasingly shallow placements within the U-shaped backbone.

#### 2.3.1 LiDeOCTNet-E

The terminal encoding block alone is converted into a deformable block, with its two ordinary 3 × 3 convolutions replaced by two 3 × 3 deformable kernels (Figure 2a).

#### 2.3.2 LiDeOCTNet-ED1

Both the terminal encoder block and the leading decoder block are converted into deformable blocks, yielding four deformable kernels in total spread across the two blocks (Figure 2c). At this depth, the spatial dimensions of the feature maps are at their minimum.

#### 2.3.3 LiDeOCTNet-ED2

Deformable blocks occupy the intermediate resolution level of both the encoding and decoding branches (Figure 2d), with each block again containing two 3 × 3 deformable kernels.

#### 2.3.4 LiDeOCTNet-ED3

Deformable blocks are positioned at the outermost resolution stage of both the encoder and the decoder (Figure 2e), where the spatial dimensions of the feature representations are at their maximum.

### 2.4 Dataset

We assess LiDeOCTNet on three OCT datasets that span retinal and endoscopic acquisitions: the AROI retinal dataset (7), an *in-vivo* sheep airway dataset (6), and a rat colon endoscopy dataset (5). Taken together, these collections cover multiple anatomical sites, biological species, and imaging hardware, and so provide a stringent and varied evaluation setting. As Figure 3 illustrates, the three datasets share the defining property that all tissue layers are demarcated by undulating interfaces extending across the full lateral span of each scan.

**Figure 3:**
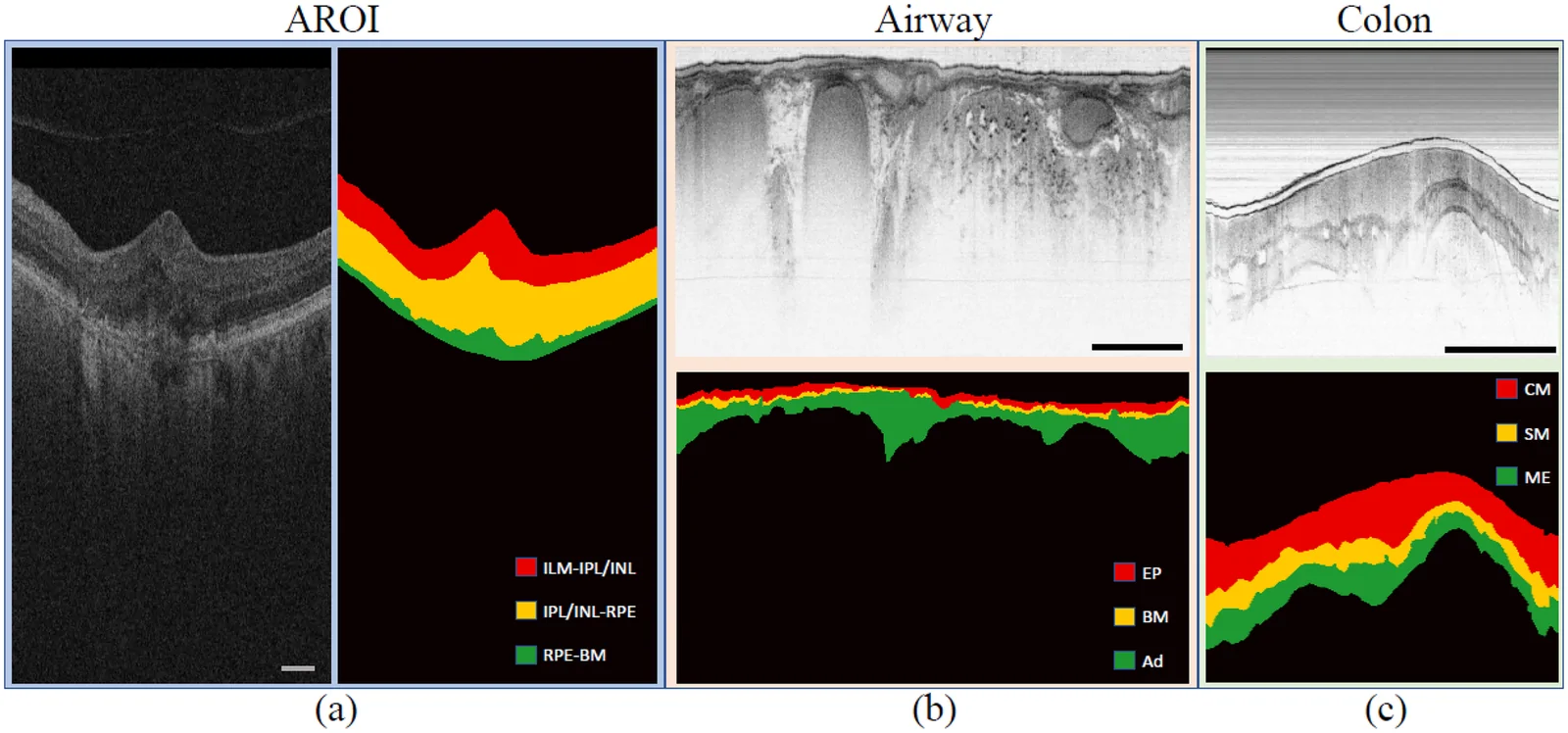
Representative OCT scans and their layer annotations from the three datasets used in this study. Across all three, the tissue strata exhibit undulating, non-flat boundaries that traverse the entire lateral extent of each B-scan. **(a)** Retinal (AROI) scans (7; Source: AROI database), with a pixel pitch of 1.96 × 11.74 µm. **(b)** Airway scans at a pixel pitch of 1.7 × 7.2 µm. **(c)** Colon scans at a pixel pitch of 1.7 × 6.8 µm. The annotation covers three retinal regions, namely the strip between ILM and IPL/INL, the strip between IPL/INL and RPE, and the strip between RPE and BM. **(b)** Airway scans at a pixel pitch of 1.7 × 7.2 μm. Three layers of the airway wall are labelled: epithelium (EP), basement membrane (BM), and adventitia (AD). **(c)** Colon scans at a pixel pitch of 1.7 × 6.8 μm, showing colonic mucosa (CM), submucosa (SM), and muscularis externa (ME). Image intensities were standardised within each dataset prior to training in order to mitigate the strong contrast between tissue and background. Scale bars: 1 mm.

#### 2.4.1 AROI dataset

The AROI collection (7) comprises volumetric OCT acquisitions from patients with neovascular age-related macular degeneration (nAMD), recorded on a Zeiss Cirrus HD-OCT 4,000 device. Every 3D volume holds 128 B-scans of 1,024 × 512 pixels at a pixel pitch of 1.96 × 11.74 µm, an example of which appears in Figure 3a. Three retinal regions are manually labelled: the band running from the internal limiting membrane (ILM) to the boundary between inner plexiform and inner nuclear layers (IPL/INL); the band reaching from IPL/INL to the retinal pigment epithelium (RPE); and the band stretching from RPE to Bruch's membrane (BM). The 1,136 annotated B-scans were partitioned at random into 796 for training, 170 for validation, and 170 for testing.

#### 2.4.2 Airway dataset

The airway dataset originates from *in-vivo* recordings on anaesthetised sheep, obtained with an 800 nm ultrahigh-resolution spectral-domain OCT system. Only airways whose internal luminal diameter was below 1 mm were imaged, an example of which is shown in Figure 3b (6). Three layers of the airway wall received annotations: epithelium (EP), basement membrane (BM), and adventitia (AD). The collection holds 418 labelled images of 1,408 × 2,326 pixels at a pitch of 1.7 × 7.2 µm. A random split assigned 346 to training, 36 to validation, and 36 to the test set.

#### 2.4.3 Colon dataset

The colon dataset comprises rat colonic tissue captured with an 800 nm ultrahigh-resolution endoscopic spectral-domain OCT system (5). Three colonic wall layers were annotated, namely colonic mucosa (CM), submucosa (SM), and muscularis externa (ME), and an example appears in Figure 3c. The native acquisitions are 2,048 ×  2,326 pixels with a pixel pitch of 1.7 × 6.8 µm. The 144 labelled scans were divided into 120 training, 12 validation, and 12 test images.

### 2.5 Pre-processing

Prior to training, AROI scans were resized to 512 × 256 pixels, airway scans to 256 × 512 pixels, and colon scans to 256 × 256 pixels. The variation of aspect ratio across the three collections doubles as an indirect test of how robustly LiDeOCTNet handles different image geometries. Intensity values were then standardised separately per dataset using Equation (2):

$$p{}^{\prime }\;=\;(\;p\;-\;\mu^{D})\;/\;\sigma^{D}$$ (2)

in which µ^D^ and *σ*^D^ are the mean and standard deviation of the pixel intensities across dataset *D*, while *p* and *p*′ are the original and standardised intensity values, respectively. Normalising the intensity distribution in this way accelerates and stabilises convergence. The training-time augmentation set comprised random horizontal flips and random crop-with-zoom up to 5%. We deliberately omitted vertical flips, because reflecting an OCT image along the axial axis would invert the anatomical stacking order and thus produce physically implausible inputs. The crop-with-zoom transformations were chosen to emulate the minor positional variations introduced by probe placement.

### 2.6 Evaluation metrics

Two complementary measures are adopted to quantify segmentation quality. The first is the Dice similarity coefficient, which captures the spatial overlap between the predicted mask and the ground-truth mask, weighting correctly identified foreground pixels more heavily. Within our setup, true positives (TP) are pixels correctly assigned to a target class, false positives (FP) are pixels mislabelled as that class, and false negatives (FN) are pixels of that class that the network misclassified into a different category. The Dice coefficient is then expressed as Equation (3):

Dice=2×TP/(2×TP+FN+FP) (3)

The second measure is the Hausdorff distance (HD), which quantifies how faithfully the model reproduces the location of tissue boundaries [M3]. Rather than treating every pixel equally, HD examines only those that lie on or near the boundary, scoring the spatial discrepancy between the predicted contour and the reference contour. We adopt the average formulation of HD, which integrates over all boundary point pairs rather than reporting only the worst-case offset, as given by Equation (4):

$$HD(Gt,\;Pd)\;=\;mean_{\;p_{\;pd}\in Pd}\;min{\;}_{\;p_{gt}\in Gt}|\;p_{gt}\;-\;p_{\;pd}{|}^{2}$$ (4)

in which Pd and Gt denote the predicted and ground-truth boundary point sets, $p_{\;pd}$ is a point on the predicted boundary, and $p_{gt}\;$ is its nearest counterpart on the reference boundary. Read together, the Dice coefficient summarises region-level overlap while the Hausdorff distance summarises boundary-level fidelity, and the two metrics jointly provide a comprehensive view of model performance.

### 2.7 Training details

All variants of the network were realised in PyTorch and trained on a workstation equipped with two NVIDIA RTX 3090 GPUs (24 GB each). Optimisation employed stochastic gradient descent (SGD) with mini-batches of eight images, an initial learning rate of 0.0004, and a momentum coefficient of 0.9. We applied a patience-based learning-rate schedule: when the validation metric showed no improvement over ten consecutive epochs, the learning rate was scaled down by a factor of ten, and training was halted after four such reductions had been triggered. In practice, most runs converged within roughly 100–120 epochs, although a handful concluded near 80 epochs and a few extended to about 200. The training loss combined a standard cross-entropy term with a class-weighted mean-squared-error (MSE) regulariser, as given by Equation (5):

$$L\;=\;-log(\;pG)\;+\;w(G\;-\;P{)}^{2}$$ (5)

in which pG denotes the softmax probability that the network assigns to the ground-truth class, *G* and *P* are the ground-truth and predicted class labels, and *w* is a per-class weighting factor that counters the impact of class imbalance. The class-weight assignments were {1, 1, 1, 3} for AROI (background, ILM–IPL/INL, IPL/INL–RPE, RPE–BM), {1, 2, 5, 1} for the airway dataset (background, EP, BM, AD), and {1, 1, 2, 2} for the colon dataset (background, CM, SM, ME). Section 4.4 provides the rationale behind these specific values.

## 3 Experiments and results

### 3.1 Effect of deformable kernel location

All five LiDeOCTNet variants (Figure 2) were trained and evaluated on identical data partitions to determine the most effective placement for the deformable block. The learnt offset fields are visualised in Figure 4, with the corresponding quantitative numbers gathered in Table 1. Inspecting the offsets in Figures 4a–c reveals that, in LiDeOCTNet-E and LiDeOCTNet-ED1, the kernels at the deepest network level acquire markedly larger spatial offsets than those situated at shallower levels. The decoder-side deformable kernels in LiDeOCTNet-D show a smaller, but still appreciable, degree of deformation compared with their encoder-side counterparts in LiDeOCTNet-E, and they nevertheless outperform every variant where deformable kernels are confined to higher resolution levels (Table 1). At the opposite extreme, kernels at the shallowest stage display almost no spatial offset (Figures 4d,e). LiDeOCTNet-ED3, accordingly, fails to segment at least one tissue class on every dataset, with Dice values below 0.5 in all cases that render the corresponding HD measurement uninformative (Table 1). Considered as a whole, these observations consistently support the view that larger kernel deformation aids tissue-layer discrimination in both retinal and endoscopic OCT, and that the deepest network level is the natural place to permit such deformation.

**Figure 4:**
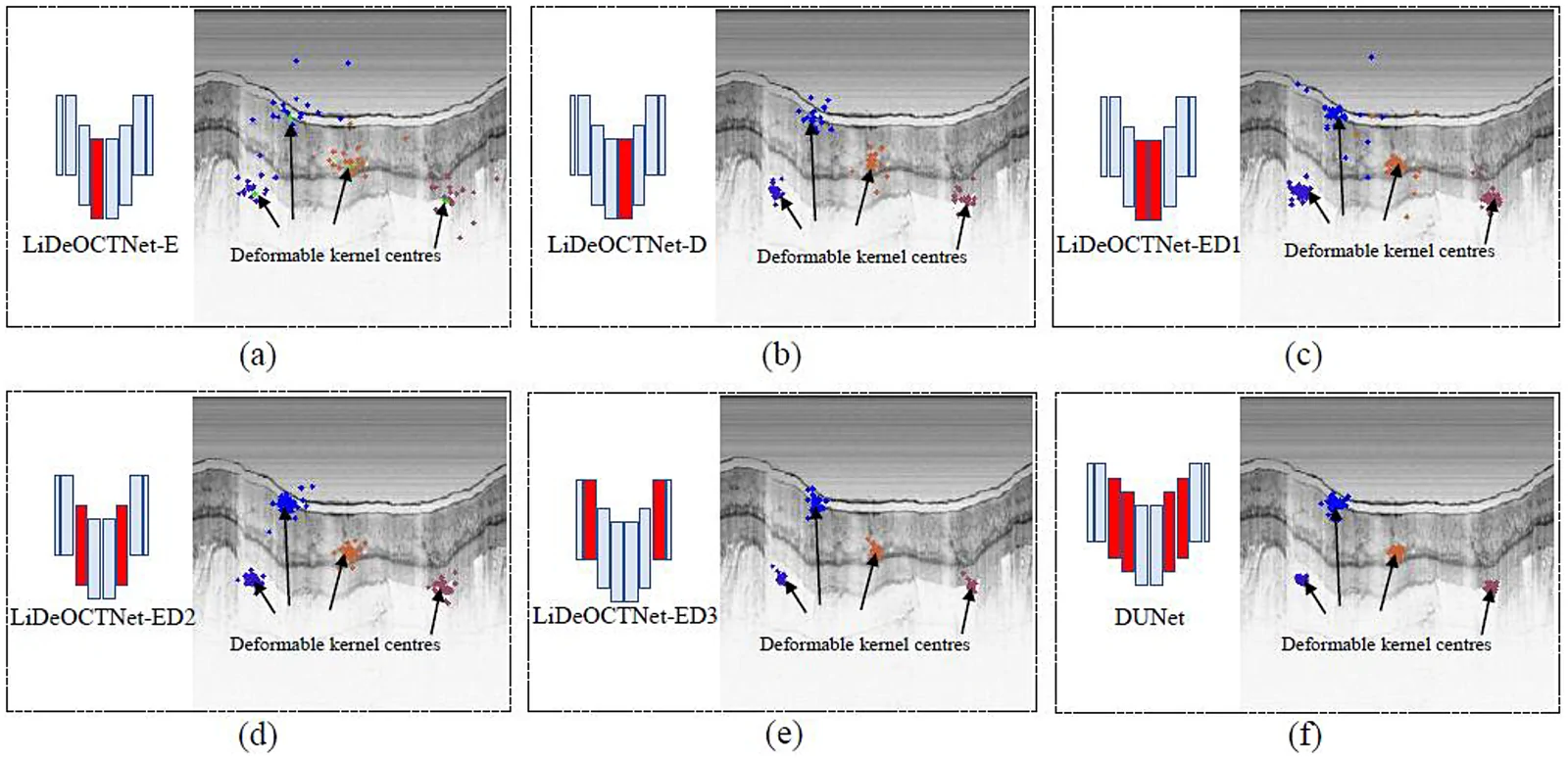
Visualisation of the learnt kernel offsets at selected spatial positions for each LiDeOCTNet variant, evaluated on the colon dataset. Configurations whose deformable blocks lie at the deepest level **(a,b,c)** yield substantially larger offsets than those positioned at shallower levels **(d,e,f)**. The wider deformation enables a single kernel to span several irregular layers simultaneously, which manifests as the quantitative gains reported in Table 1.

**Table 1:** Segmentation accuracy (dice and average hausdorff distance) and computational cost for every LiDeOCTNet variant and DUNet on the three OCT datasets.

Network	AROI Dice	AROI HD	Airway Dice	Airway HD	Colon Dice	Colon HD	Params	Inf. time
LiDeOCTNet-E	0.9607	1.448	0.9257	2.501	0.9198	2.152	0.14M	8.20
LiDeOCTNet-D	0.9605	1.519	0.9160	2.875	0.9115	2.479	0.15M	14.78
LiDeOCTNet-ED1	0.8586	8.274	0.8779	7.394	0.8923	4.679	0.17M	44.17
LiDeOCTNet-ED2	0.6446	10.601	0.7178	13.448	0.7205	9.551	0.15M	35.41
LiDeOCTNet-ED3	0.2272	—	0.2986	—	0.2141	—	0.13M	30.40
DUNet	0.9709	0.925	0.9367	2.021	0.9080	2.650	0.88M	61.56

DUNet edges ahead of LiDeOCTNet-E on two datasets, but at more than six times the parameter count. (Red: highest score; blue: runner-up.).

DUNet (47) originally designed for retinal vessel segmentation, employs a four-level backbone with deformable blocks at its two intermediate resolution stages. Within our comparison, LiDeOCTNet-ED2 mirrors this placement scheme, but it fails to match the performance of LiDeOCTNet-ED1 (Table 1), in which the deformable blocks reside at deeper levels. Although DUNet edges past LiDeOCTNet on a few metrics [Figure 4(f)], it does so at the cost of more than six times as many parameters as LiDeOCTNet-E. Distributing deformable kernels across four blocks, in the manner of DUNet, therefore yields only marginal benefit over the simpler strategy of concentrating one deformable block at the deepest encoder stage. On this basis, LiDeOCTNet-E was chosen as the reference configuration for the remainder of the experiments, since it attains near-optimal performance with the smallest parameter budget.

### 3.2 Effect of network size

The dependence of segmentation performance on model capacity was probed by sweeping the base channel width *b* of the three-level UNet backbone over the range *b* = 4 to *b* = 24. Adjusting *b* uniformly rescales the channel count at every stage of the network. Both LiDeOCTNet-E and the standard UNet were evaluated across this range, which was selected because the metrics for both architectures plateau at the upper end, signalling diminishing returns from additional channel width. Each architecture–width pair was trained from three independent random initialisations; runs in which the model collapsed to zero recall on at least one tissue class were discarded, and per-metric mean and standard deviation were reported across the surviving runs.

A small number of runs at very low-capacity exhibited training instability, manifesting as zero recall on one or more classes. By systematically removing such failed runs, we expose the functional range of each architecture, that is, the smallest parameter budget at which the model dependably learns every annotated class. Figures 5, 6 show how Dice and HD evolve with parameter count. On the AROI and airway data, LiDeOCTNet-E retains stable performance at notably smaller sizes than the standard UNet: for AROI, LiDeOCTNet-E becomes stable around 70 k parameters whereas UNet requires more than 110 k; for the airway dataset, the corresponding thresholds are roughly 130 k and 160 k. On colon data, the two architectures plateau at a similar threshold near 70 k. More importantly, at every parameter count where both architectures yield valid segmentations, LiDeOCTNet-E delivers a higher Dice and a lower HD. In practical terms, a more compact LiDeOCTNet-E can take the place of a larger conventional UNet without conceding accuracy, often with measurable improvements. These traits are especially attractive for embedding the network into real-time OCT processing pipelines.

**Figure 5:**
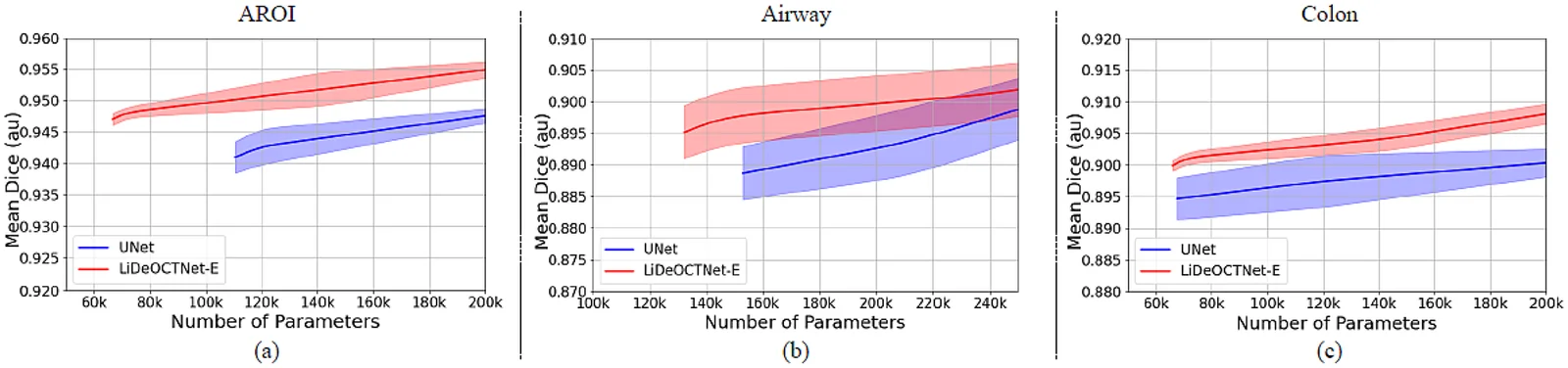
Dice score plotted against parameter count for LiDeOCTNet-E and UNet on **(a)** AROI, **(b)** airway, and **(c)** colon data. Solid lines show the mean across three independent training runs, while shaded bands indicate ±1 standard deviation. Across every parameter setting tested, LiDeOCTNet-E sits above the plain UNet, demonstrating that a smaller deformable model can stand in for a larger fixed-kernel one with no loss of accuracy.

**Figure 6:**
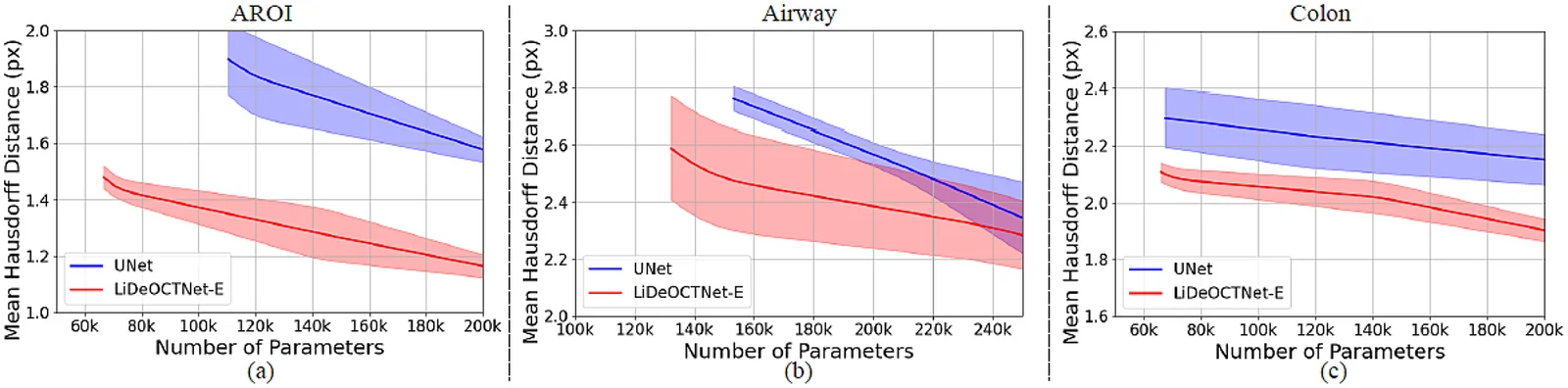
Average hausdorff distance plotted against parameter count for LiDeOCTNet-E and UNet on **(a)** AROI, **(b)** airway, and **(c)** colon data, with curves and shaded regions following the convention of Figure 5. The boundary-level advantage of LiDeOCTNet-E mirrors the area-level advantage seen in the Dice plots and persists across every tested network size.

Qualitative outputs at b = 12 (around 120 k parameters) appear in Figure 7. Across all three datasets, including the geometrically demanding colon scans (see Table 3 for a quantitative description of dataset difficulty), LiDeOCTNet-E delineates tissue boundaries more crisply than the standard UNet. Boundary-localisation errors made by UNet are highlighted in the figure with white circles. UNet additionally produces topological errors, in which portions of one tissue layer are misclassified as a neighbouring class; this failure mode is most apparent in the airway images (white squares, Figure 7). LiDeOCTNet-E largely escapes these errors, owing to the spatially adaptive receptive fields of its deformable kernels, which retune their sampling pattern to match the local tissue geometry.

**Figure 7:**
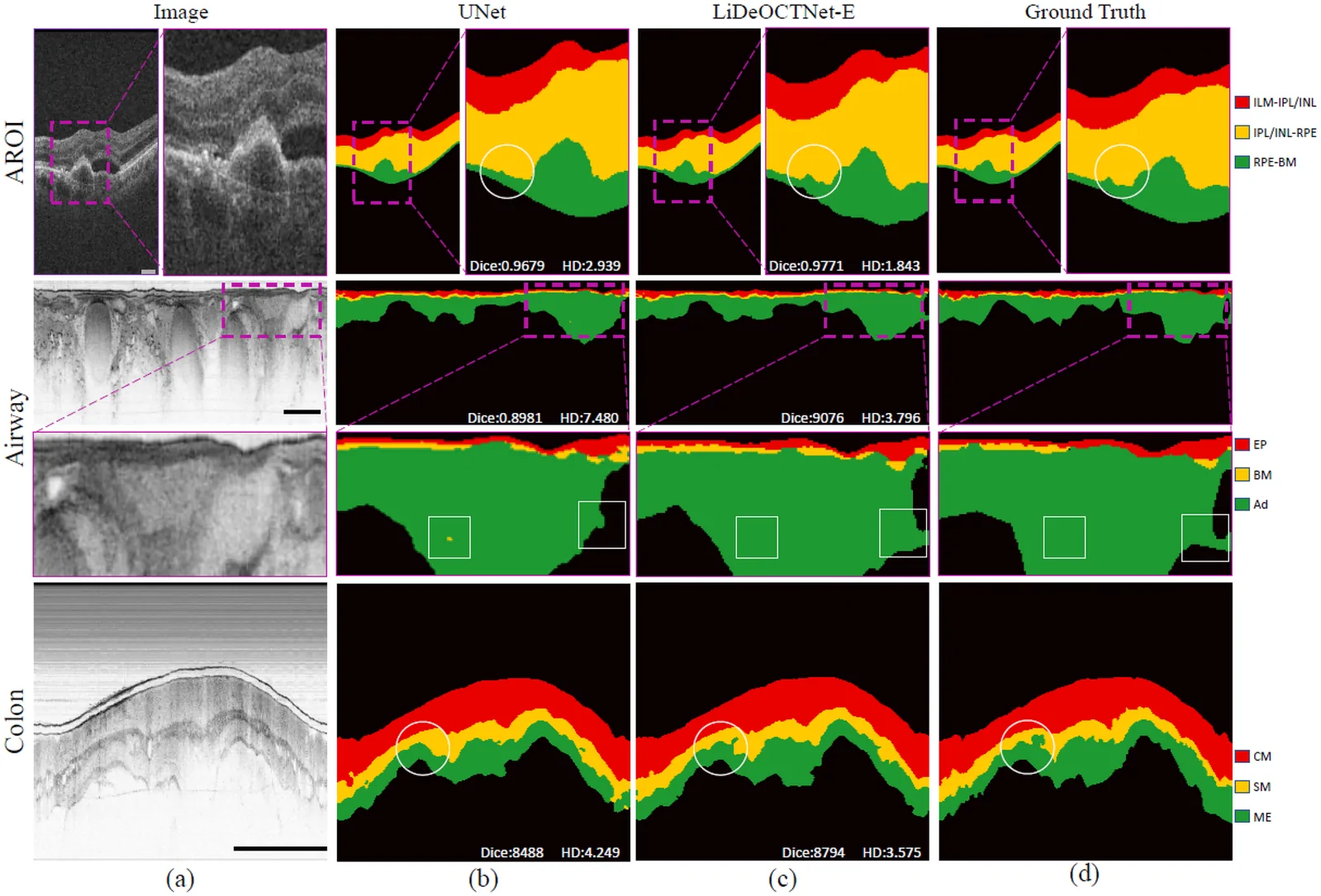
Illustrates segmentation results on representative AROI, airway, and colon test images. Columns show **(a)** the input image, **(b)** UNet segmentation, **(c)** LiDeOCTNet-E segmentation, and **(d)** ground-truth annotations. Despite using only a single deformable block, LiDeOCTNet-E generates contours that more closely follow the ground truth at both coarse and fine scales. White circles indicate locations where UNet deviates from the true boundary, and white squares mark interior regions where UNet assigns incorrect layer labels—errors that LiDeOCTNet-E largely avoids. Scale bars: 1 mm.

### 3.3 Comparison with state-of-the-art networks

LiDeOCTNet-E was benchmarked against three established reference architectures: TransUNet (35), which augments a UNet-style decoder with a transformer-based encoder; DefEDNet (48), in which deformable convolutions populate the entire encoding pathway; and BlazeNeo (49), a lightweight design built on the HarDNet backbone. Each of these models has demonstrated strong performance on a range of medical image segmentation benchmarks. To keep the comparison fair, every reference network was sourced from its publicly released codebase and retrained under the experimental protocol described in Section 2.7. The only modification we applied was to adapt the input layer to single-channel grey-scale images in place of the three-channel RGB input the original models expect.

Representative predictions from all four models appear in Figure 8. Of the four, TransUNet reproduces the irregular tissue boundaries most faithfully, a result that fits well with the ability of self-attention to capture extended spatial dependencies across an image (34, 35). Nevertheless, LiDeOCTNet-E also generates visually plausible predictions that align closely with the ground truth, and in many regions it is on par with, or better than, both DefEDNet and BlazeNeo.

**Figure 8:**
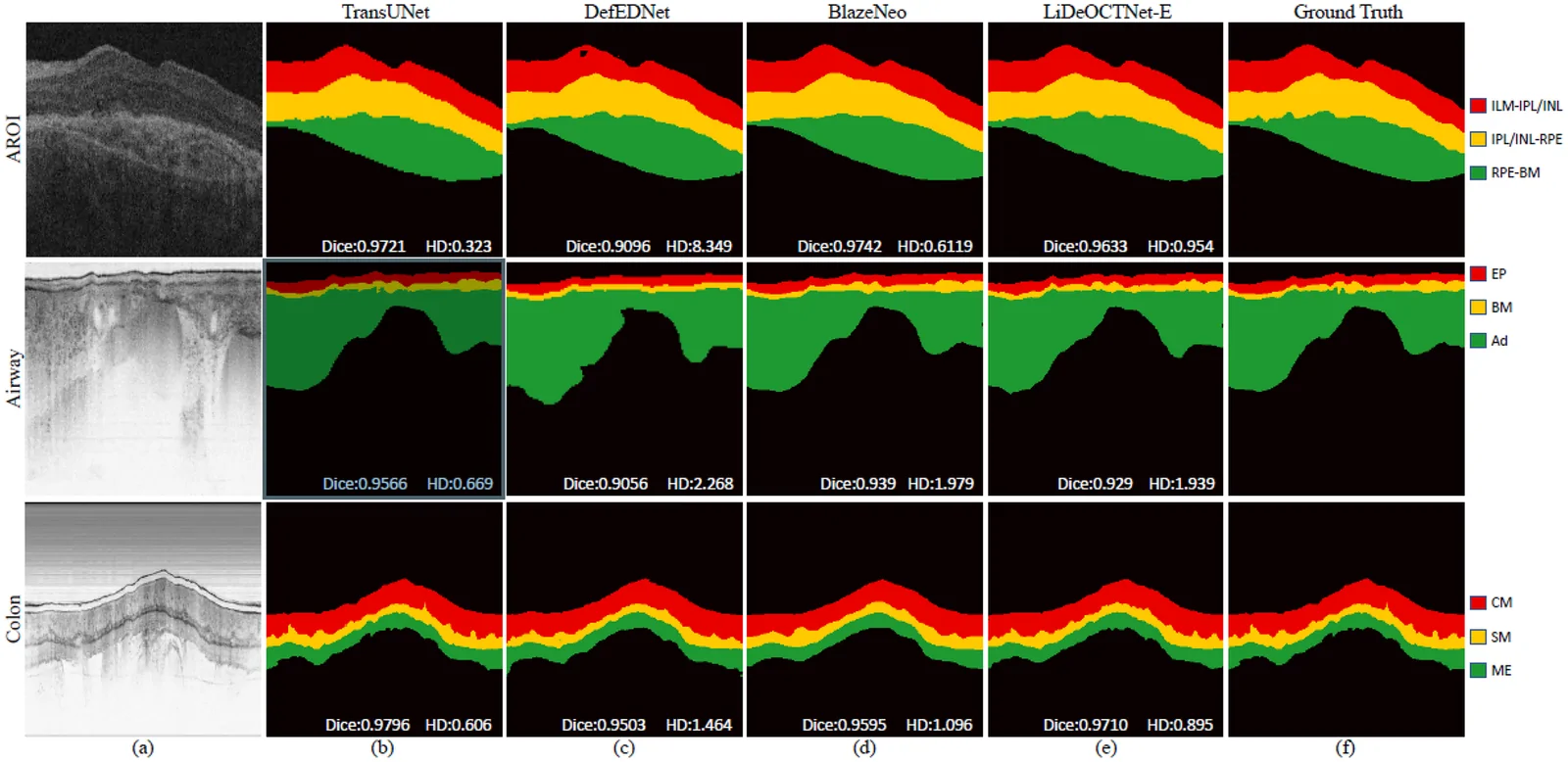
Qualitative comparison of LiDeOCTNet-E with three state-of-the-art networks on representative test scans drawn from the AROI, airway, and colon datasets: **(a)** input scan, **(b)** TransUNet output, **(c)** DefEDNet output, **(d)** blazeNeo output, **(e)** LiDeOCTNet-E output, and **(f)** ground-truth annotation. The segmentations produced by LiDeOCTNet-E closely resemble those of the substantially heavier baselines, with Dice deviations typically inside ∼1% and HD deviations inside 1 pixel; the airway dataset constitutes a partial exception, owing to its more pronounced class imbalance.

The numbers in Table 2 corroborate this visual impression. On the colon dataset, the most geometrically demanding of the three (Table 3), LiDeOCTNet-E records a Dice of 0.9198 and an HD of 2.152, beating DefEDNet and BlazeNeo and trailing TransUNet only marginally (Dice 0.9265, HD 2.080). Yet TransUNet uses 105.3 M parameters versus 0.14 M for LiDeOCTNet-E, and runs roughly 7.4 ×  slower. On the AROI and colon collections, the Dice gap between LiDeOCTNet-E and the top performer is approximately one percentage point (1.12% on AROI and 0.67% on colon), with HD gaps of 1.030 and 0.072 pixels, respectively. The gap widens on the airway dataset (2.75% Dice, 1.229 HD pixels), in line with that dataset showing a lower boundary irregularity index (Table 3). Put differently, LiDeOCTNet-E performs best, relative to the comparison baselines, precisely in the scenarios in which tissue boundaries are most tortuous, which is exactly the regime that deformable kernels were designed to address.

**Table 2:** Quantitative comparison of LiDeOCTNet-E against TransUNet, DefEDNet, and blazeNeo on the three OCT datasets.

Network	AROI Dice	AROI HD	Airway Dice	Airway HD	Colon Dice	Colon HD	Params	Inf. time
LiDeOCTNet-E	0.9607	1.448	0.9257	2.501	0.9198	2.152	0.14M	8.20
TransUNet	0.9719	0.418	0.9532	1.272	0.9265	2.080	105.3M	60.57
DefEDNet	0.8410	8.947	0.8301	4.682	0.9042	2.877	13.33M	88.64
BlazeNeo	0.9701	0.937	0.9464	1.337	0.8969	2.898	6.17M	40.12

Despite using orders of magnitude fewer parameters and recording the lowest inference time, LiDeOCTNet-E achieves Dice and HD scores that remain closely competitive with the best-performing baselines. (Red: top score; blue: runner-up.).

**Table 3:** Dataset-level complexity analysis. The proposed metric fuses boundary irregularity (*α*) and class imbalance (*β*) into a single composite score.

Dataset	Dimensions (Network input)	*Α*	Mean *α*	SD *α*	*β*	Complexity
AROI	1,024 × 512 (512 × 256)	1.079 1.218 1.536	1.278	0.191	0.933	1.072
Airway	1,408 × 2,326 (256 × 512)	1.025 0.978 1.600	1.201	0.2830	0.943	1.044
Colon	2,048 × 2,326 (256 × 256)	1.374 1.472 1.429	1.425	0.0399	0.916	1.129

Higher complexity is associated with lower segmentation accuracy across every tested network: AROI is the easiest, colon the hardest, and airway lies in between, in agreement with the quantitative results presented in Tables 1, 2.

## 4 Discussion

### 4.1 Task-specific location of deformable kernels

Where the deformable blocks are placed in the network has a strong influence on segmentation quality, and the optimal location is conditional on the imaging modality being addressed (47). Our experiments indicate that, although stacking multiple deformable blocks may bring incremental gains, a single block at the deepest encoder stage already accounts for most of the achievable improvement; adding a counterpart block at the leading decoder stage contributes only a minor extra benefit (Figure 4). We attribute this pattern to the horizontally banded structure of OCT images: a kernel whose receptive field is wide enough to span several tissue layers simultaneously is uniquely positioned to recognise which layer hosts its centre. Balancing the very small parameter count against the modest run-to-run variation, LiDeOCTNet-E with deformable kernels concentrated inside the deepest encoder block emerges as the recommended configuration for practical OCT segmentation deployments.

### 4.2 Lightweight networks

Both UNet and LiDeOCTNet experience a sharp performance breakdown once the parameter count drops below a critical threshold, with one or more tissue classes failing to be learnt at all (Figures 5, 6). At low capacities, LiDeOCTNet is generally more reliable than the standard UNet, although the LiDeOCTNet-ED1 variant is the exception: it is less stable than either LiDeOCTNet-E or LiDeOCTNet-D (Table 1) and yields lower accuracy. At the upper end of the capacity range, networks of every architecture, including the plain UNet, converge on broadly similar accuracy levels. The low-capacity regime, however, is the region of most interest for real-time deployment (55–57) A long-standing principle in efficient network design (58–60) is to concentrate the most expensive operations at the spatial bottleneck, where the feature maps are at their smallest. LiDeOCTNet adheres to this principle by restricting its deformable operations to the deepest, most spatially compressed level of the network. The same principle is also evident in recent lightweight OCT designs such as LightReSeg (41) and LKMU-Lite (42), both of which prioritise compactness while preserving segmentation accuracy (Table 4).

**Table 4:** Comparison of lightweight segmentation models across the three datasets, reporting parameter count.

Model	Year	Params (M)	Param ratio
LiDeOCTNet-E (ours)	—	0.14	1.0 ×
EGE-UNet	2023	0.053	0.38 ×
ESEUNet-ESK	2023	0.222	1.59 ×
CMUNeXt-S	2024	0.418	2.99 ×
CGNet	2020	0.492	3.51 ×
ULite	2023	0.878	6.27 ×
VM-UNet (Mamba)	2024	∼1.1	∼7.9 ×

Our proposed network LiDeOCTNet-E matches or exceeds competing lightweight networks at a fraction of the parameters.

A direct comparison of the MAC counts required by each model is provided in Figure 9. Because the offset prediction inside a deformable kernel does not entail floating-point multiplication, deformable networks accumulate fewer floating-point operations than fixed-kernel networks at the same parameter count. With b held constant, deformable models do incur a small MAC overhead from the offset-prediction subnetwork; once we instead match models on total parameter count, however, LiDeOCTNet variants require fewer MACs than the corresponding UNet (modulo the integer constraint on b, which prevents an exact match at every size). This further reinforces the argument that compact LiDeOCTNet configurations are well-suited to deployment alongside high-throughput OCT instruments. The fact that networks below 2.0×10n MACs can still segment irregular tissue boundaries accurately is a direct consequence of the kernel-node repositioning mechanism.

**Figure 9:**
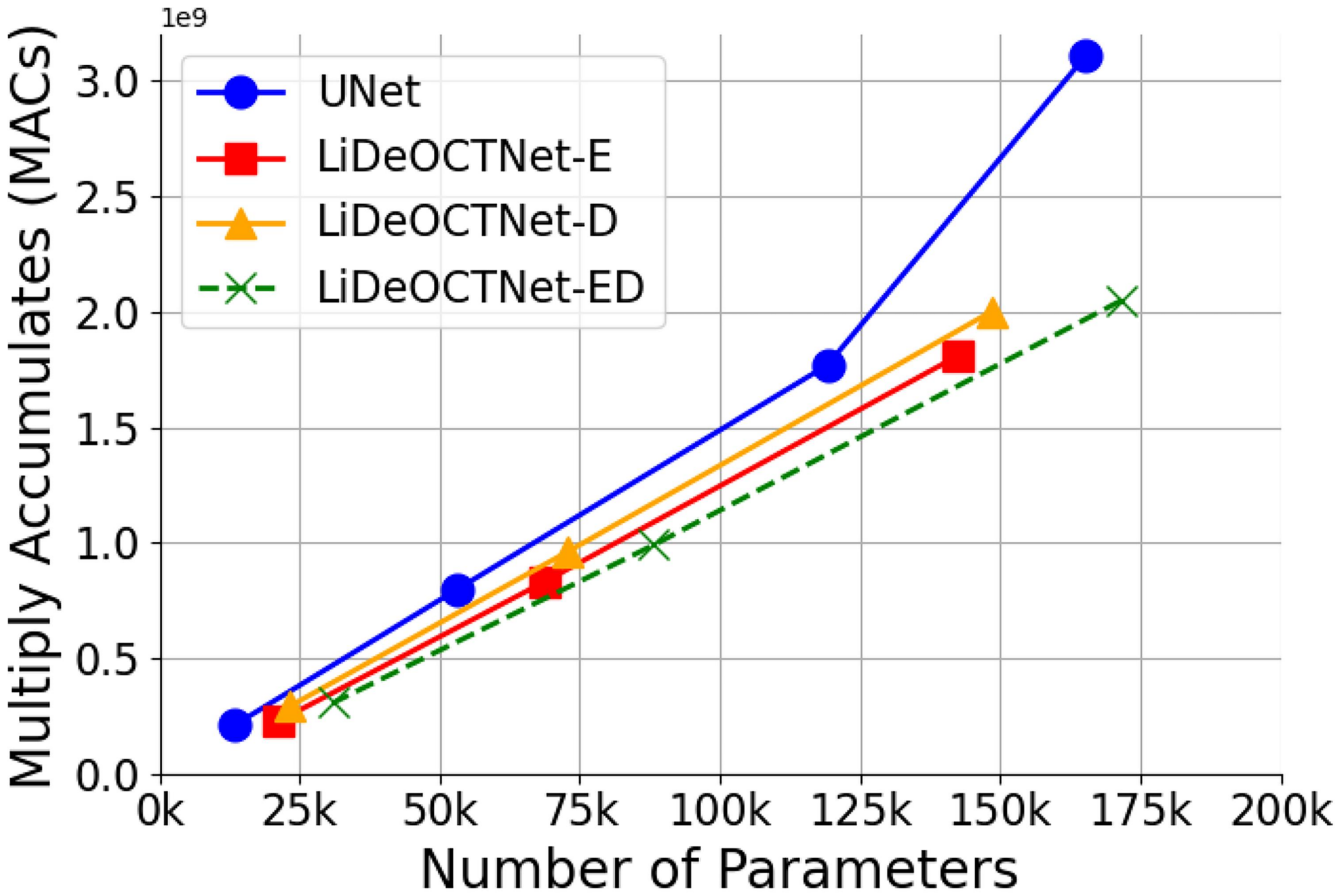
Multiply-accumulate operations (MACs) plotted against parameter count for UNet and the LiDeOCTNet family, with each marker corresponding to a particular base-channel setting b. At equivalent parameter budgets, LiDeOCTNet variants demand fewer MACs than the standard UNet, an attribute that favours their adoption in latency-sensitive OCT workflows.

Three orthogonal directions exist for further trimming the computational footprint of LiDeOCTNet: shrinking the number of network levels, narrowing the channel widths, and lowering the input resolution. Complementary compression techniques such as structured channel pruning (59) and reduced-precision arithmetic [M8] could in principle be combined with the present design to yield further compactness. We leave their exploration to future work.

Figure 10 provides an overall summary of the efficiency–accuracy trade-off across all benchmarked networks. LiDeOCTNet-E lies inside the preferred operating zone, where high mean Dice and low parameter count are achieved together. This is the region most relevant to high-throughput clinical use.

**Figure 10:**
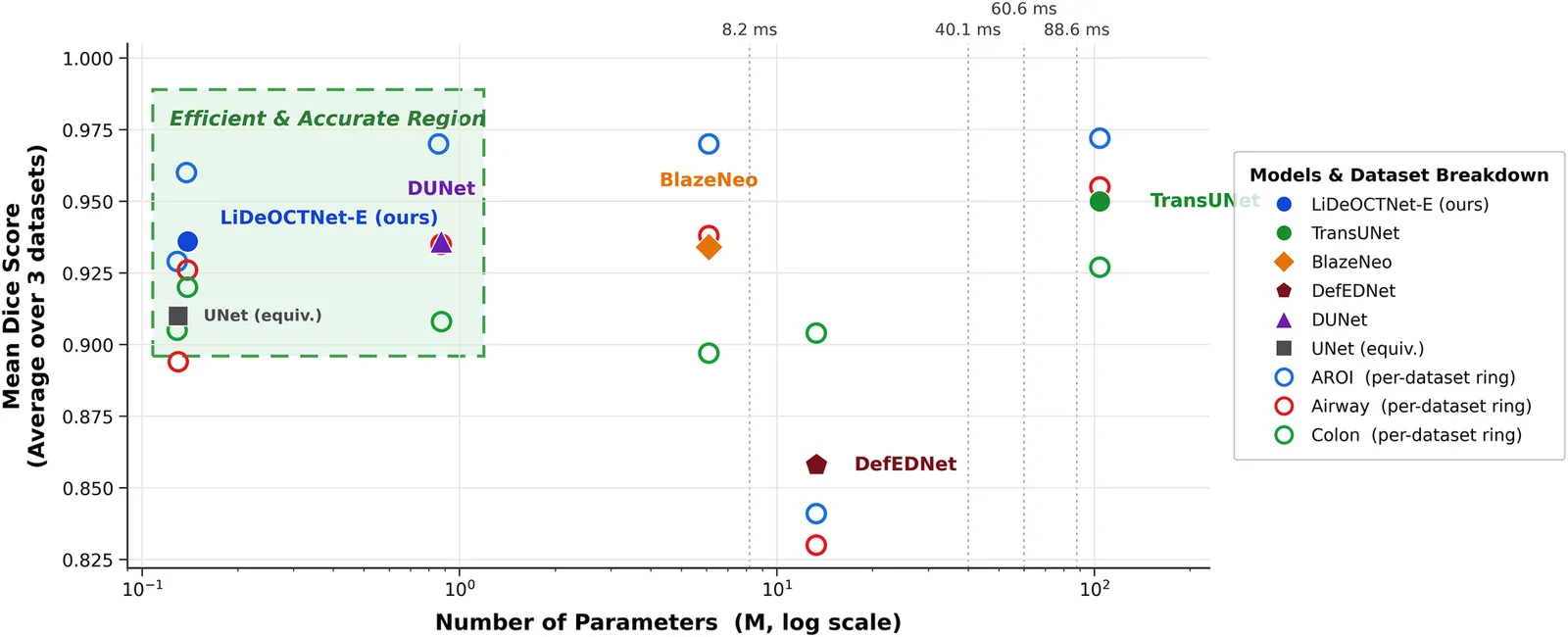
Efficiency–accuracy trade-off for all evaluated networks. The horizontal axis shows the total number of trainable parameters **(M)** on a logarithmic scale; the vertical axis shows the mean Dice score averaged across the three OCT datasets. Large filled markers represent dataset-averaged scores; hollow coloured rings at the same horizontal position show per-dataset breakdowns (blue: AROI, red: Airway, green: Colon) for LiDeOCTNet-E and TransUNet. Vertical dashed lines indicate inference latency milestones. The shaded green region denotes the preferred operating zone, combining high accuracy with a low parameter count. LiDeOCTNet-E (solid blue circle) sits squarely within this zone, achieving a mean Dice within 1.5% of TransUNet while using ∼750 ×  fewer parameters and operating ∼7.4 ×  faster.

### 4.3 OCT data complexity analysis

To enable a principled comparison across the three datasets, we devised a complexity index that simultaneously quantifies the two main difficulties of OCT segmentation: tissue-boundary tortuosity and inter-class pixel-area imbalance. The index is defined as Equation (6):

$Complexity=w1(|α−1|)+w2⋅β$ (6)

in which *α* denotes the ratio between the actual arc length of a tissue boundary and the image width, thereby quantifying boundary waviness, while *β* = 1−average class proportion captures how far the per-class pixel distribution departs from a uniform allocation. The weights w₁ = 0.5 and w₂ = 1 encode the estimated relative contribution of each factor to overall difficulty. Per-layer values of *α*, alongside summary statistics (mean and standard deviation of *α*), the value of *β*, and the resulting composite complexity score, are reported in Table 3.

The parameter *α* captures how far tissue boundaries diverge from an idealised straight horizontal baseline. The colon dataset records the highest mean *α* value (1.425) and consequently the highest overall complexity score, which aligns with the observation that every tested model, LiDeOCTNet-E included, attains its weakest scores on this dataset (Table 2). On the colon data, the two architectures equipped with deformable kernels LiDeOCTNet-E and DefEDNet exhibit a relatively larger advantage over non-deformable methods than they do on the other datasets. This is consistent with the interpretation that spatially adaptive sampling is most useful precisely when boundaries are most irregular, an insight that is plausibly applicable beyond OCT to broader medical image analysis and computer vision. A similar trend has been reported in recent lightweight OCT studies that pair attention mechanisms with adaptive feature extraction (41, 43).

Class imbalance, captured by *β*, contributes an independent source of difficulty (7). Although AROI has a higher boundary-roughness index than the airway data, it nevertheless yields the better segmentation scores. The discrepancy can be traced back to the airway dataset's more pronounced class imbalance (*β* = 0.943 versus 0.933 for AROI; Table 3). The same imbalance is also responsible for the largest gap between LiDeOCTNet-E and the leading competitor occurring on the airway dataset (Table 2): LiDeOCTNet-E was designed primarily to address boundary irregularity rather than severe class skew.

Supplementary analyses of contrast and signal-to-noise ratio (SNR) across the three datasets revealed no consistent association with the observed inter-dataset variation in segmentation quality, suggesting that the per-dataset intensity standardisation effectively neutralises their contribution. The proposed complexity index therefore retains only boundary tortuosity and class imbalance as the explanatory factors behind the cross-dataset performance pattern.

### 4.4 Failure cases and limitation

Figure 11 points to two situations where our method still struggles. The first is the edge case where a tissue layer slowly fades into the background. In these regions the signal is so weak. The human annotators also face problem to guess where the true boundary sits or not. Since there is no clear intensity change for the deformable kernels to lock onto, the predicted contour can drift away from the manual label. The second problem shows up when the tissue has many small, continuous “holes” packed into a tiny area, as shown in rat colon images. In such cases the shape changes too quickly and over too fine a scale for the offset field to follow. In this case, the deformation cannot fully wrap around all of these tight gaps. As a result the model tends to smooth over the fine detail and misses part of the true layer shape.

**Figure 11:**
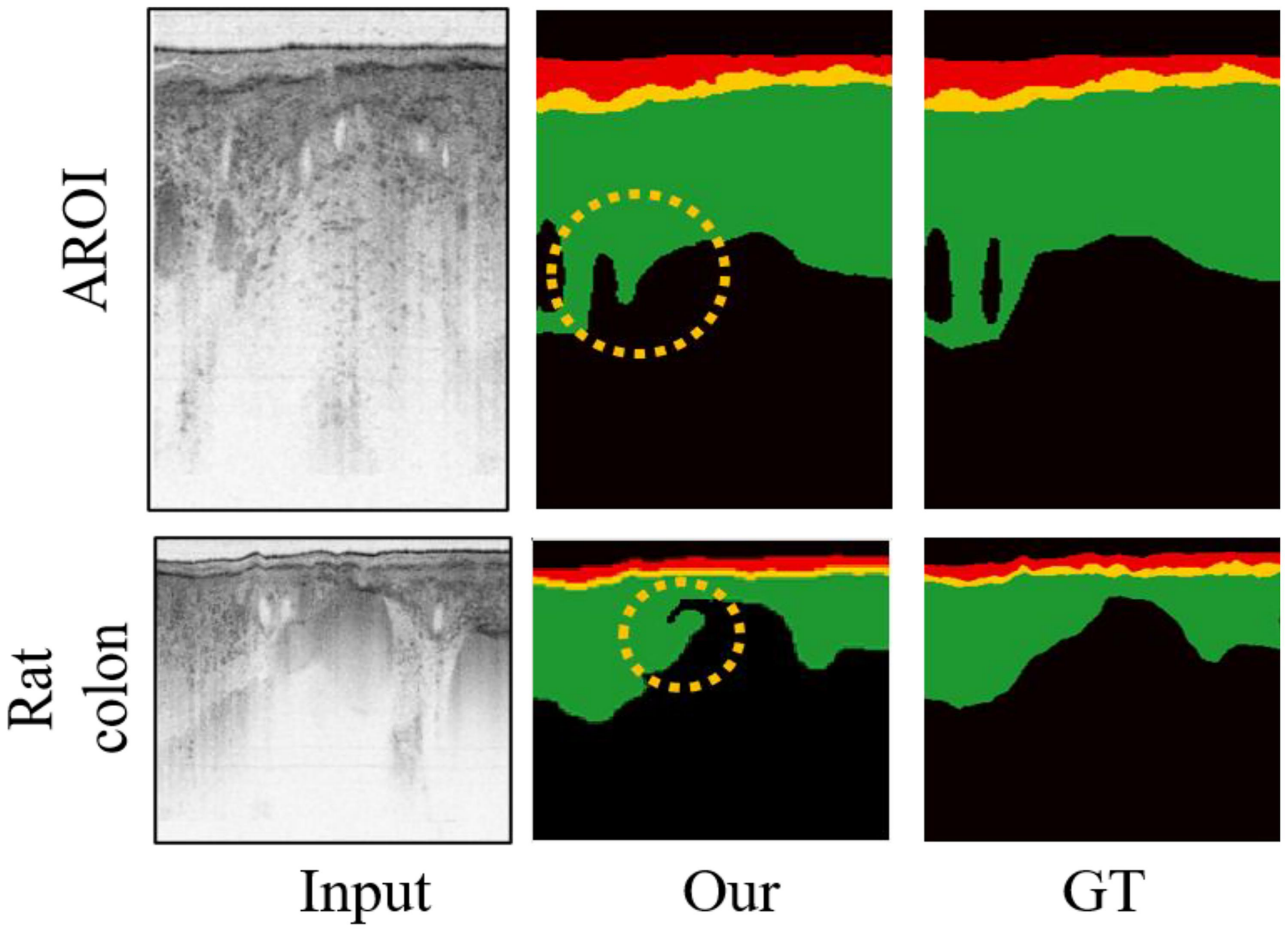
Through our experiments, a challenging area of the proposed network is identified as twofold: 1. The edge case where the layer fades into the back ground, and the manual labelling used human reasoning to delineate the most likely location of the boundary. 2. When there are continuous “holes' in a small area, the deformation may not be able to fully capture the shape of the layers.

### 4.5 Loss function

The choice of training objective shapes the learning dynamics and ultimately the quality of the learned segmentation function. After a systematic evaluation of candidates from both distribution-based and region-based loss families (61), cross-entropy was identified as the most stable baseline. We then augmented it with a class-weighted MSE penalty, as defined in Equation (5). The MSE component imposes a soft regularisation constraint that encourages the network to respect the anatomically ordered, stratified tissue organisation typical of OCT data. Each class receives a weight that reflects its relative importance during optimisation. Initial weight values were derived from the empirical pixel frequency distributions in the training images and then refined through pilot experiments.

Several design principles emerged from this process. First, assigning the background class a lower weight than the tissue classes hurt foreground–background discrimination; we therefore fixed the background weight at unity, and at least the most prevalent tissue class was assigned the same weight. Second, less prevalent tissue classes were given higher weights, roughly in proportion to how underrepresented they are in pixel area. Third, classes with greater boundary tortuosity, as indicated by elevated *α* values in Table 3, received additional weight increments based on the pilot run outcomes.

For AROI, the weights for {ILM–IPL/INL, IPL/INL–RPE, RPE–BM} were {1, 1, 3}: the first two layers occupy comparable pixel areas, while the RPE–BM stratum is thinner and harder to segment, which justifies the higher weight. For the airway dataset, the weights for {EP, BM, AD} were {2, 5, 1}: both EP and BM are spatially more limited than AD, and BM has an *α* value below unity, which means it appears as discontinuous segments in some acquisitions and is much harder to segment, justifying the weight of 5. For the colon dataset, the weights for {CM, SM, ME} were {1, 2, 2}: the three layers occupy broadly similar areas, but SM and ME have more tortuous boundaries than CM, which calls for a moderate weight increase. A full grid search over the weight space would probably yield further improvements; however, the values above were sufficient to support a fair comparison across all evaluated architectures. Crucially, the same class weights were applied to every network in the comparative study, so that no model receives a differential advantage from the loss tuning.

## 5 Conclusion

This paper has presented LiDeOCTNet, a deliberately compact segmentation backbone in which a slimmed-down UNet is paired with strategically positioned deformable convolution kernels for the delineation of irregular tissue strata in OCT scans. By keeping the parameter budget low without compromising segmentation fidelity, the network lends itself naturally to deployment in resource-constrained clinical environments where low latency is essential. Our experiments consistently single out the deepest encoder stage as the most effective insertion site for deformable kernels in the OCT setting, although this conclusion may need re-examination when the design is transferred to other imaging modalities. Across both ophthalmic and endoscopic OCT collections, LiDeOCTNet attains results that are quantitatively and visually competitive with much heavier and more elaborate baselines, while operating at noticeably higher throughput. Alongside the network itself, we proposed a dataset complexity index that gives a principled handle on segmentation difficulty in terms of boundary tortuosity and class-area imbalance, and we used this index to inform the design of the loss function. Future research will examine additional avenues for improving segmentation accuracy, computational efficiency, and cross-dataset generalisation, with the broader aim of supporting reliable, real-time use of LiDeOCTNet in routine clinical practice.

## Data Availability

The original contributions presented in the study are included in the article/Supplementary Material, further inquiries can be directed to the corresponding authors.
